# Prevalence of a virus similar to human hepatitis B virus in swine

**DOI:** 10.1186/1743-422X-7-60

**Published:** 2010-03-17

**Authors:** Wengui Li, Ruiping She, Liqiang Liu, Hua You, Jun Yin

**Affiliations:** 1 College of Veterinary Medicine, China Agricultural University, Beijing 100193, China; 2 College of Animal Science and Technology, Yunnan Agricultural University, Kunming 650201, China; 3 College of Agriculture, Hebei University of Engineering, Handan 056021, China

## Abstract

**Background:**

The objective of this study is to established evidence of the existence of a novel member of the hepadnavirus family endemic in swine. Temporarily this virus was designated as swine hepatitis B virus (SHBV). This SHBV can be detected by using human hepatitis B virus diagnostic kits including ELISA, immunohistochemical staining, and transmission electron microscopy (TEM). Also seroprevalence of pig farms in Beijing, China, and pathological features of SHBV infection was determined.

**Results:**

Screened result shows that overall prevalence of HBsAg was 24.8%, closed to that of anti-HBsAg, whereas HBeAg and anti-HBe were barely detectable. The distribution of HBsAg and HBcAg was examined by immunohistochemistry of liver samples. Typical hepatitis pathological change, such as spotty parenchymal cell degeneration, necrosis of hepatocytes and proliferation of fibrous connective tissue were observed during histopathological analysis. Analysis of HBsAg-positive serum with TEM revealed two morphologic forms, 20 nm and 40 nm sized particles, similar to small spherical and Danes particles of HBV. Observation of the ultrastructure of the liver also found HBV-like particles in the nucleus of hepatocytes.

**Conclusion:**

Our research result implies that SHBV could be a causative agent of swine. The discovery of SHBV will unveil novel evolutionary aspects of hepatitis and provides new information for further hepadnavirus research.

## Background

Viral hepatitis B remain a serious medical challenge worldwide [1]. A strong epidemiological relationship has been established between persistent hepatitis B virus (HBV) infection and hepatocellular carcinoma (HCC) [2]. HBV is one of the smallest enveloped animal viruses with a virion diameter of 42 nm. But pleomorphic forms exist, including filamentous and spherical bodies lacking a core. As most hepadnaviruses, HBV will only replicate in specific hosts, and this makes experiments using in vitro methods very difficult.

Formerly, hepatitis B was called serum hepatitis. Detection of HBV infection involves serum or blood tests that detect either viral antigens (surface antigen HBsAg and e antigen HBeAg) and antibodies (anti-HBs, anti-HBc, anti-HBe), known as HBV serological marker. HBsAg is most frequently used to screen for the presence of this infection, the presence of HBeAg in a host's serum is associated with much higher rates of viral replication and enhanced infectivity. Nevertheless, interpretation of these assays is complex.

HBV is the prototype member of a steadily growing family of hepadnaviruses which can be found in both mammals (orthohepadnaviruses) and birds (avihepadnaviruses). Orthohepadnaviruses have been identified so far in woodchucks (WHV), ground and arctic squirrels (GSHV, ASHV), and primates including woolly monkeys (WMHBV), orangutans, gorillas, and gibbons [3-8]. Avihepadnavirus has been reported in various duck species (DHBV), grey herons (HHBV), geese (GHBV), Ross's goose (RGHBV), storks (STHBV), and cranes (CHBV) [9-11]. The discovery of HBV-related viruses offers ample opportunities for *in vivo *studies of various animals with naturally occurring hepadnaviruses. This has been valuable in determining the mechanisms of hepadnavirus replication, pathogenesis of hepatocellular carcinoma (HCC), and for antiviral drug studies.

HBV-related hepadnaviruses in mammalian and avian species has been valuable in HBV studies. Like determining the mechanisms of hepadnavirus replication, pathogenesis of HCC, and antiviral drug studies [12]. However, most of the corresponding animals are difficult to handle in captivity or not easily available. Since none of the currently available animal models are ideal, the development of additional experimental animal models promises to provide answers for many HBV research questions [13].

Researchers have concentrated on a group of HBV-like viruses in domestic animals since 1985 [14]. Using human HBV diagnostic kits, a number of domestic animals are positive for HBV serological marker [15,16], electron microscope observed HBV-like virion in HBsAg positive serum of swine, Holstein, cattle, canine and sheep; even gene sequence highly homologous to HBV has been amplified [17-20]. Nevertheless, Up to the present time, none of these HBV-like viruses been systematically identified and related reports found only in China. Here we characterize the prevalence of HBV-like virus in swine which may provide an interesting model for comparative studies of liver pathology and cancer associated with chronic hepadnavirus infections.

## Results

### Enzyme-linked immunosorbent assay

**Figure 1:**
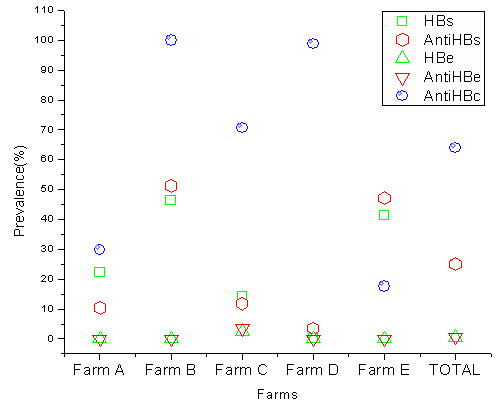
**Prevalence of SHBV serological markers among 416 swine sera samples collected from five farms in Beijing, China**. Scatter graphs showed that nearly a quarter of the swine have been infected by SHBV. Prevalence rates of HBs were close to anti-HBs, while HBeAg and anti-HBe were hardly detected.

To investigate the current prevalence of SHBV in swine herds, 416 samples of swine serum collected from 5 randomly selected farms in Beijing, China, were tested for HBV serological markers using a commercial ELISA kit. Briefly, overall prevalence of HBsAg was 24.8%, and profoundly close to anti-HBs (25.0%), while HBe and anti-HBe was hardly detected (0.5% and 0.7%), indicating no common antigen existed in HBe. The overall prevalence of anti-HBc was 63.9% (Fig. 1, Table 1).

**Table 1:** Prevalence of SHBV serological markers among 416 swine sera samples collected from five farms

	n	HBsAg, *n *(%)	HBsAb n (%)	HBeAg, *n *(%)	Anti-HBe, n (%)	Anti-HBc, n (%)
Farm A	77	17 (22.1)	8 (10.4)	0 (0)	0 (0)	23 (29.9)
Farm B	84	39 (46.4)	43 (51.2)	0 (0)	0 (0)	84 (100)
Farm C	85	12 (14.1)	10 (11.8)	2 (2.4)	3 (3.5)	60 (70.6)
Farm D	85	0 (0)	3 (3.5)	0 (0)	0 (0)	84 (98.8)
Farm E	85	35 (41.2)	40 (47.1)	0 (0)	0 (0)	15 (17.6)

Total	416	103 (24.8)	104 (25.0)	2 (0.5)	3 (0.7)	266 (63.9)

### Histopathological analysis and Mallory's trichrome stain

**Figure 2:**
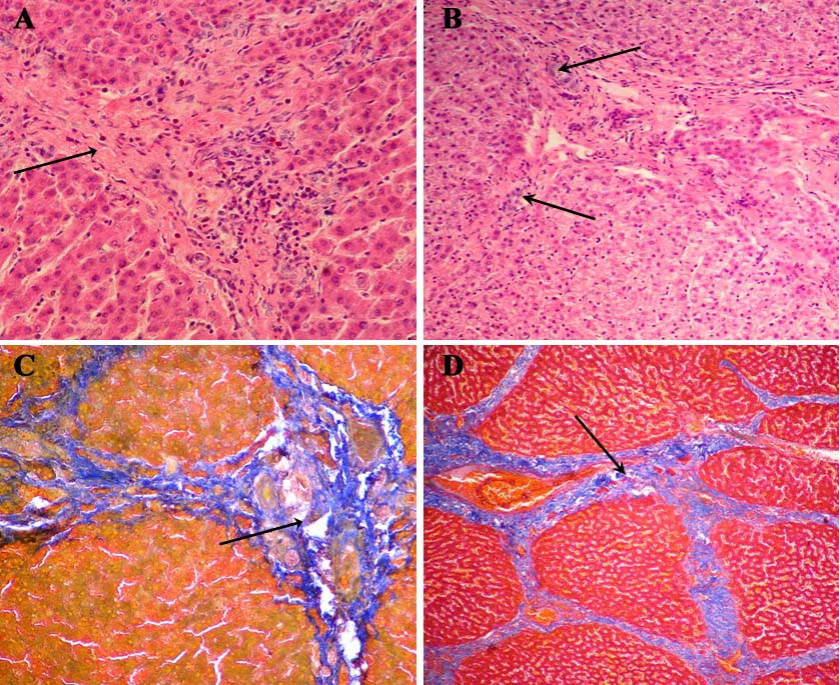
**Results of histopathological analysis(A, B) and Mallory's trichrome stain(C, D)**. (A) desmoplasia between hepatic lobule (arrow), (B) infiltration of lymphocytes (down arrow), hyperplasy of bile canaliculus and fibrous tissue at portal area(up arrow), also coagulation necrosis and karyopyknosis of hepatocytes could be seen. Original magnification × 400. (C, D) Showing proliferation of connective tissue between liver lobule (arrow). Mallory staining method, Original magnifications ×200. (A, C: liver sample from CP74; B, D: liver sample from DX385).

For swine CP74 and DX385, although obvious pathological changes were not observed at autopsy, pathological changes were observed under light microscope. Gross histopathological findings showed desmoplasia in hepatic lobules, infiltration of lymphocytes, hyperplasy of bile canaliculus, and fibrous tissue at the portal area (Fig. 2A and 2B). Severe fibrous connective tissue proliferation was observed by Mallory staining (Fig. 2C and 2D). In contrast, no obvious changes were found in liver tissues collected from swine CP59.

### Immunohistochemistry

**Figure 3:**
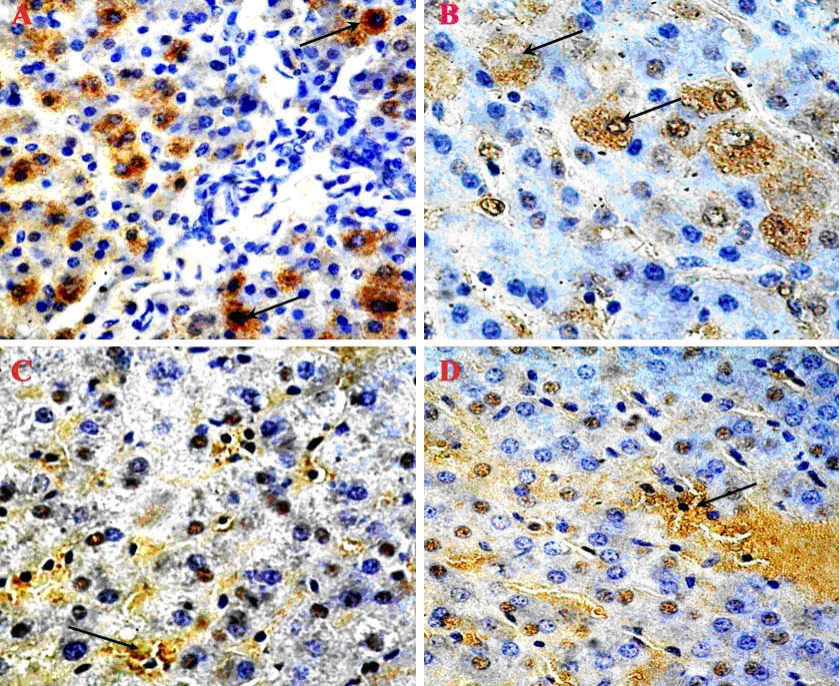
**Immunohistochemical analysis of HBsAg and HBcAg in liver tissues**.(A) Strong HBsAg immune positivity was shown in hepatocytes (arrow). (B) Immunopositivity for HBsAg was mainly distributed in cytoplasm of hepatocytes. (C, D) HBcAg was distributed mainly distributed nucleus of hepatocytes. Spotty parenchymal cell degeneration, with necrosis and karyopyknosis (arrow) of hepatocytes were observed. Original magnification×400. (A, C: liver sample from CP74; B, D: liver sample from DX385).

Immunohistochemical scanning of expression of viral antigens found that liver tissues from both swine contained HBsAg and HBcAg. Strong immunohistochemical signal was seen within hepatitis lesions. HBsAg was detected in the nucleus and cytoplasm of hepatocytes, while HBcAg was mainly distributed in the nucleus of hepatocytes. Necrosis as karyorrhexis, pyknosis and karyolisis was observed in immunohistochemically positive hepatocytes. This indicates that SHBV was pathogenic to swine, and replication of SHBV caused the necrosis of hepatocytes directly (Fig. 3A, B, C and 3D).

### Detection of viral particles in swine sera and liver cells by electron microscopy

**Figure 4:**
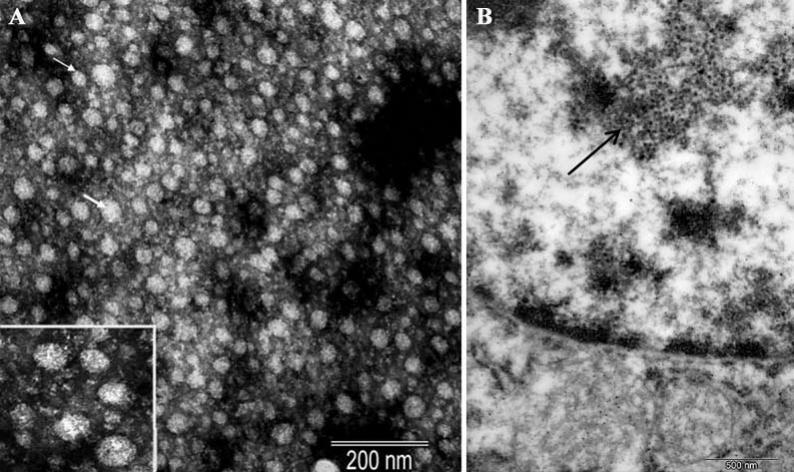
**Viral particles in swine sera and hepatocytes revealed by electron microscopy**. A: Electron micrographs of negatively stained SHBV particles from HBsAg positive serum. Two types of particles were observed which are similar in size (20 nm and 40 nm) and morphology, like complete and empty viral particles of SHBV. B: Virus-like particles in the nucleus of hepatocytes (liver sample from DX385).

To obtain ultrastructural evidence for the presence of HBV-related viral particles in the swine sera containing S antigen, HBsAg-positive serum was collected, viral particles in the sera of infected swine were morphologically analyzed by electron microscopy and sera negative for HBsAg served as controls. Essentially, two types of particles closely resembled in size (20 nm and 40 nm) and morphology, like complete and empty viral particles of HBV, were observed. However, it is puzzling that no tubular particles were seen. Particles were observed only in serum positive for HBsAg, and the number of 40 nm particles was much more than expected (Fig. 4A). Ultrastructurally, HBV-like particles were observed in the nucleus of hepatocytes (Fig. 4B).

## Discussion

Serological diagnosis of hepatitis B virus infection relies on a combination of qualitative assay results and different patterns are representative of acute or chronic disease in a carrier [21]. By examining the antigen-antibody system, hepatitis B infection is diagnosed, the course of the disease is observed and treatment is monitored [22]. The screening of HBV serological markers in swine herds showed that nearly a quarter of swine have been infected. However, profiles in SHBV serology were quite different from human HBV (data not shown). Anti-HBc is found in all people infected with HBV, which can persist for many years and act as a lifelong marker of hepatitis B [23]. The high prevalence of anti-HBc in swine (63.9%) may indicate that these swine have a history of infection. Nevertheless, existence of anti-HBc as the only serological marker also may be the result of nonspecific cross-reaction with other agents [24].

Though hepadnaviruses are host specific, HBV infections also occur frequently in chimpanzee, gibbon and other ape populations in sub-Saharan Africa and South-East Asia where the HBV infection rate in apes was remarkably comparable to that of human population in these areas [25,26]. Scientists are concerned about the ability of HBV to cross species barriers. Large reservoirs of infection in apes may hamper ongoing attempts to permanently eradicate HBV infection from the human population through immunization [27].

The prevalence of HBV among human and the nonhuman primates maybe speed up the evolution process. Due to high error rate of the viral reverse transcriptase, and recombination among different genotypes or hepadnavirus strains from human and nonhuman primates, the eight genotypes of HBV have further diverged into at least 24 subgenotypes, with certainly many more still to be identified [28]. Interspecies recombination events of HBV also occur among human and nonhuman primates [29], such as gibbons of different genera, chimpanzees, and birds of different subfamilies [25,30]. Interspecies recombination of hepadnaviruses from cross-species hosts would provide a large variation in virus genomes, which would change pathogenecity and transmissibility, and expand the host range. Evidence for recombination of human and ape HBV variants demonstrates that human and nonhuman-associated HBV variants can indeed share hosts in nature [30]. Compared to nonhuman primates, domestic animals are in more contact with humans and the possibility of interspecies recombination is higher. Thus the discovery of SHBV will be beneficial to research of HBV evolution.

The lack of suitable in vitro infection systems and appropriate animal models has hampered the progress of HBV research, but progress has been made through the identification of avian and mammalian HBV-related viruses. However, none of these natural hosts are commonly used laboratory animals, and the expense and difficulty in handling these animals have limited their usage [4,31,32]. In fact, chimpanzees are the only animals fully permissive and well tested for HBV infection. Nonetheless, the limited availability and the high cost of keeping primates severely restricts their use in research [13]. Comparatively, pigs are widely used in medical research and there are abundant in supply, since the available animal models are not ideal, the development of additional experimental animal systems is warranted, the finding of HBV in pigs will enhance our understanding of the virology and immunology of HBV infection and disease pathogenesis, including major sequelae like chronic hepatitis and hepatocellular carcinoma.

Of the 350 million to 400 million individuals worldwide infected with the hepatitis B virus (HBV), one-third reside in China, with 130 million carriers and 30 million chronically infected [33]. Even though a vaccination program for newborn babies has been in place since the 1990s, the incidence of hepatitis B is still increasing, from 21.9 in 100,000 people in 1990 to 53.3 in 100,000 in 2003. The reason for this increased HBV infection is unknown, because hepatitis B has no clear transmission routes in many people in China [34]. The identification of the SHBV strain confirms that a novel class of hepadnaviridae exists in swine populations. And thus brings about a lot of questions. Does these pigs infected by HBV? Does swine hepadnavirus exist? Does this virus related to the rising of hepatitis B in human population? But before these questions could be answered, further studies are needed to elucidate the structure, assembly, genome organization and regulation of gene expression of this novel hepadnavirus.

## Methods

### Swine and serum samples

To determine the seroprevalence of SHBV infection in swine, 416 swine serum samples were collected from five randomly selected farms in Beijing, China. For serum collection, 5 mL of blood was collected from swine into dry tubes. After clotting and centrifugation, sera were separated and stored at -20°C until use. Two swine positive for HBsAg (CP74:Boar, 7 month; DX385: Sow, 8.5 month) were sacrificed to determine the possible relationship of SHBV infection and histopathological changes in the liver. Another swine negative for all serological markers (CP59:Sow, 5 month) was sacrificed and served as a negative control.

### Serological analysis of hepatitis B virus markers

All serum samples were screened for hepatitis B serological markers (anti-HBc, HBsAg, anti-HBs, HBeAg, and anti-HBe) with a commercial enzyme-linked immunosorbent assay (ELISA) kit (SIIC Kinghaw Biotech Co. Ltd., Beijing, China) according to the manufacturer's recommendations. The absorbance was determined at 450 nm (Multiscan Titertek MCC). Blank, negative and positive controls were included on each plate. Data were analyzed with the SPSS software for Windows (SPSS Inc., Chicago, USA) and a scatter graph was obtained by using OriginPro 7.5 (OriginLab Corporation, Northampton, MA, USA).

### Histopathology analysis and Mallory's trichrome stain

Histopathological analysis was used to study the pathological characteristics of SHBV infection. In consideration of fibrosis is the pathological feature of chronic hepatitis, Mallory trichrome stain was used to study fibrous tissue proliferation in liver. Liver samples were collected and fixed in 2.5% (v/v) glutaraldehyde-polyoxymethylene solution immediately after swine were sacrificed. The tissue samples were dehydrated and embedded in paraffin. Sections of 5-μm thickness were then prepared for hematoxylin and eosin (H&E), and Mallory trichrome stains. For Mallory's trichrome stain, paraffin sections were washed with distilled water and immersed in 3% dichromicum kalium for 5 min, then in solution consisting of 0.1% acid fuchsin for 2 min, and 0.5% aniline blue for 20 min. Thereafter the slides were washed sequentially with distilled water, 95% ethanol, and three changes of 100% xylene. After the xylene had evaporated, Cytoseal 60 mounting medium was applied, and the slides were coverslipped for examination under a microscope. All powdered stains used for Mallory stain were obtained from Sigma (Sigma Co., Beijing, China).

### Immunohistochemistry

Serial paraffin sections (5 μm) were prepared and kept at 37°C for more than 12 hours. The sections were immersed in three consecutive washings in xylol for 5 min to remove paraffin, and then hydrated through graded alcohol. Sections were incubated for 30 min and blocked with 3% peroxide at room temperature for endogenous peroxidase ablation. The following steps were carried out in a moist chamber. Sections were incubated with blocking buffer (Zymed Laboratories Inc., San Diego, USA) containing 20% normal goat serum (Gibco) and 80% PBS (0.01 M, pH 7.4) at 37°C for 30 min. After discarding the goat serum, sections were incubated in primary monoclonal antibodies against HBsAg and HBcAg (Zhongshan Golden Bridge Biotech Co. Ltd., Beijing, China) diluted in PBS, for 2 hours at 37°C. After rinsing for 3 times in PBS-T, sections were incubated with the goat anti-mouse IgG conjugated with HRP (Sigma) at 37°C for 1 hour and rinsed 3 times in PBS-T. The specimens were incubated with 3,3-diaminobenzidin (DAB; Zymed Laboratories Inc) at room temperature for 10 min in the dark. Finally, sections were stained with hematoxylin for 8 min after rinsing for 3 times in PBS-T, dehydrated, and mounted with neutral gums. Sections for the negative control group were prepared by the same steps as described above but with the HBsAg and HBcAg antibodies replaced by PBS.

### Detection of viral particles in swine sera and hepatocytes by transmission electron microscopy

To obtain ultrastructural evidence for the presence of HBV-related viral particles in swine sera containing S antigen, HBsAg-positive serums were collected and viral particles in sera of infected swine were morphologically analyzed by electron microscopy. Sera negative for HBsAg served as controls. Serum collected from three swine were centrifuged at 4000 rpm for 10 min, then 0.01 M poly ethylene glycol 6000 (PEG6000) was added into the subsequent upper aqueous phase. After incubation overnight at 4°C, the serum was centrifuged at 20,000 rpm for 1 hour, resuspended in PBS and stained for 1 min with 1% uranyl acetate. For the thin section study, the fixative used was 2.5% paraformaldehyde-glutaraldehyde in 0.1 M cacodylate buffer (pH 7.4). The sections were postfixed in 1% OsO_4 _for 1 hour, and treated with 1% uranyl acetate, dehydrated in ethanol and embedded in Epon 812. Ultrathin sections were obtained using a routine method and stained with uranyl acetate and lead citrate. All electron micrographs were obtained with JEV1230 transmission electron microscope (JEOL Ltd., Tokyo, Japan) at 80 kV.

## Competing interests

The authors declare that they have no competing interests.

## Authors' contributions

WGL carried out the serological analysis of hepatitis B virus markers and drafted the manuscript. LQL carried out the Histopathology analysis and Mallory's trichrome stain. HY and JY carried out the immunohistochemical staining and transmission electron microscope investigations. RPS carried out the design of the study and revision of the manuscript. All authors read and approved the final manuscript.
